# Co-existence of Lumbar Disc Herniation and Posterior Ring Apophyseal Fracture: It Is Not Rare and Computed Tomography Is Useful

**DOI:** 10.7759/cureus.35475

**Published:** 2023-02-26

**Authors:** Takahiro Inoue, Akihiko Inokuchi, Teiyu Izumi, Ryuta Imamura, Takahiro Hamada, Kimitaka Nakamura, Toshihiro Ebihara, Hayato Inoue, Yosuke Kuroki, Takeshi Arizono

**Affiliations:** 1 Department of Orthopaedic Surgery, Kyushu Central Hospital of the Mutual Aid Association of Public School Teachers, Fukuoka, JPN; 2 Department of Orthopedic Surgery, Kyushu Central Hospital of the Mutual Aid Association of Public School Teachers, Fukuoka, JPN

**Keywords:** ct scan, ldh, praf, co-existence, computed tomography (ct ), endoscopic surgery, surgical outcomes research, lumbar disc herniation surgery, posterior ring apophysis fracture

## Abstract

Introduction

Posterior ring apophyseal fracture (PRAF) is characterized by the separation of bone fragments and sometimes coexists with lumbar disc herniation (LDH). However, how often these conditions coexist and the details of the clinical course remain unclear.

Methods

We analyzed 200 patients who underwent surgical treatment for LDH at our hospital from January 2016 to December 2020. Among these, we reviewed 21 patients who underwent microendoscopic surgery to treat PRAF. They consisted of 11 male and 10 female patients, ranging in age from 15 to 63 years. The average age was 32.8 months, and the average follow-up period was 39.8 years. We performed simple roentgenography and magnetic resonance imaging for all patients and computed tomography for about 80% of the patients. We evaluated the type of PRAF fragment (Takata classification), disease level, Japanese Orthopedic Association (JOA) score, Roland-Morris Disability Questionnaire (RDQ) score, operating time, intraoperative blood loss, and perioperative complications.

Results

A total of 10.5% of patients with LDH also had PRAF. The mean JOA score significantly improved from 10.6 ± 5.7 points before surgery to 21.4 ± 5.1 points at the final observation (p < 0.05). The mean RDQ score significantly improved from 17.1 ± 4.5 preoperatively to 5.5 ± 0.5 at the final observation (p < 0.05). The average operation time was 88.6 minutes. There were no complications requiring early surgery that were due to postoperative infection or epidural hematoma, but one patient required reoperation.

Conclusion

This study showed that PRAF coexisted with LDH in about 10% of cases, and the outcomes of surgical treatment were generally good. Computed tomography is recommended to improve the diagnostic rate and assist with surgical planning and intraoperative decision-making.

## Introduction

Lumbar posterior ring apophyseal fracture (PRAF) is characterized by the separation of bone fragments at the posterior rim of the superior and inferior lumbar vertebral endplates, where the ring apophysis and the adjacent vertebral body are usually incompletely fused [1,2]. PRAF is uncommon and is complicated by lumbar disc herniation (LDH) in young patients [3]. PRAF is a fissure between the fragile cartilage and bony endplates in childhood caused by acute or chronic repetitive mechanical stimulation secondary to external forces such as those occurring in sports or trauma. However, there are some theories that PRAF is a fracture of the vertebral body endplate or that it is unrelated to trauma. There is no uniform consensus on its cause [4]. PRAF is sometimes missed and is often difficult to treat. The true frequency of PRAF is difficult to estimate and easily underestimated because simple roentgenography (X-ray) and magnetic resonance imaging (MRI) cannot clearly detect lesions. Many authors have argued for surgical treatment when conservative therapy is ineffective [3,5]. Because of its rarity and diversity of classification, there is no consensus on whether decompression requires herniorrhaphy only, simultaneous fragment removal, or extensive laminectomy with fusion. [6-9]. However, the treatment options for PRAF with LDH have not yet been fully explored. This study was performed to investigate how often PRAF and LDH coexist and assess the clinical course at our hospital.

## Materials and methods

We retrospectively reviewed the data of 200 consecutive patients who underwent surgical treatment of LDH at the Kyushu Central Hospital, Fukuoka, Japan, from January 2016 to December 2020. Among them, we focused on 21 patients who underwent microendoscopic surgery to treat PRAF. The patients comprised 11 men and 10 women, ranging in age from 15 to 63 years (mean age, 39.8 years) at the time of surgery. The disease level was L4/5 in seven patients and L5/S1 in 14 patients. Takata types I, II, and III PRAF were present in three, two, and 16 patients, respectively [3]. The follow-up period ranged from 12 to 96 months (average, 32.5 months). The clinical records were analyzed for the following data: type of imaging test performed, Japanese Orthopedic Association (JOA) score, Roland-Morris Disability Questionnaire (RDQ) score, operating time, intraoperative blood loss, and perioperative complications. All statistical analyses were performed with Easy R (EZR) (Saitama Medical Center, Jichi Medical University, Saitama, Japan), which is a graphical user interface for R (The R Foundation for Statistical Computing, Vienna, Austria) [10]. Numerical variables are expressed as the mean ± standard error of the mean. The Student’s t-test was performed; if the p-value was <0.05, the null hypothesis of no difference was rejected. This study was reviewed and approved by the Institutional Ethics Committee of Kyushu Central Hospital (approval application no. 310).

## Results

PRAF was found in 21 of 200 consecutive patients (Table 1).

**Table 1:** Patient characteristics LDH: lumbar disc herniation; MED: microendoscopic discectomy; FESS: fully endoscopic spine surgery; F: female; M: male

Case	Age (in years)	Sex	Levels of LDH	Takata Classification	Surgical treatment	Bone resection
1	45	F	L4/5	3	MED	No
2	44	F	L5/S	3	MED	No
3	40	F	L5/S	3	MED	No
4	45	F	L4/5	3	MED	No
5	40	M	L5/S	2	MED	No
6	63	F	L5/S	3	MED	No
7	29	M	L4/5	1	MED	No
8	29	M	L4/5	3	MED	No
9	26	M	L5/S	3	MED	No
10	25	F	L5/S	1	MED	No
11	62	M	L5/S	3	MED	No
12	56	M	L5/S	3	MED	No
13	57	F	L4/5	3	MED	No
14	44	F	L5/S	3	MED	No
15	38	M	L5/S	3	MED	No
16	27	M	L5/S	3	MED	No
17	30	M	L5/S	3	MED	No
18	35	M	L5/S	3	MED	No
19	44	F	L5/S	3	MED	No
20	15	M	L4/5	1	MED	No
21	41	F	L4/5	2	FESS	Yes

PRAF and LDH coexisted in 10.5% of patients. X-ray and MRI were performed in all cases, whereas computed tomography (CT) was performed in about 80% of cases. The JOA score was improved after surgery in all patients. The mean score was 10.6 ± 4.67 points before surgery and 21.4 ± 5.11 points at the final follow-up (p < 0.05). The RDQ score was also improved after surgery in all patients. The mean score was 17.1 ± 4.78 points before surgery and 5.55 ± 0.55 points at the final follow-up (p < 0.05). The mean operation time was 88.6 ± 33.3 minutes. The blood loss was minimal and difficult to measure in most patients, but a few patients had a blood loss of about 100 ml. Bone fragment removal was noted in the operative record of only one patient. There were no complications requiring early surgery due to postoperative infection or epidural hematoma, but one patient required reoperation because of a recurrent hernia (Table 2).

**Table 2:** Clinical outcomes in patients with PRAF PRAF: posterior ring apophyseal fracture; JOA score: Japanese Orthopedic Association score; RDQ: Roland–Morris Disability Questionnaire

Characteristic	Outcomes
Operation time (in minutes) (Mean±SD)	88.6±4.30
Preoperative JOA score (Mean±SD)	10.6 ± 4.67
Postoperative JOA score (Mean±SD)	21.4 ± 5.11
Preoperative RDQ (Mean±SD)	17.1 ± 4.78
Postoperative RDQ (Mean±SD)	5.55 ± 0.55
Reoperation /recurrent hernia	4.76%(1/21)

## Discussion

**Figure 1:**
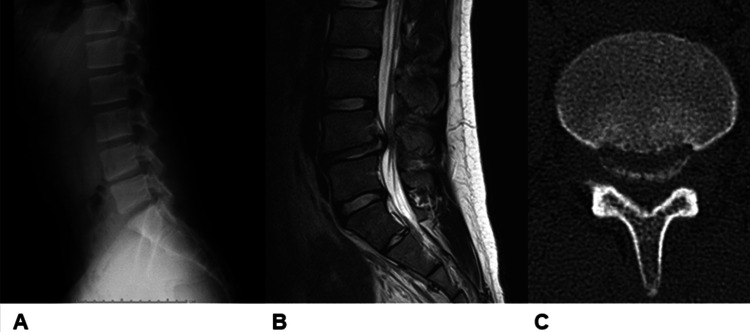
A case in which PRAF was difficult to diagnose by preoperative X-ray and MRI but could be diagnosed by a postoperative CT scan (case 20) A: A preoperative X-ray showed no obvious bone fragments; B: A preoperative MRI showed no obvious bone fragments; C: A postoperative computed tomography led to the diagnosis of Takata type I posterior ring apophyseal fracture

PRAF is rarely associated with disc herniation and is typically seen in 5% to 8% of patients with LDH [1-5]. It is more common in male patients and most common among patients in their late teens and 20s. PRAF is also seen in patients in their 30s and 40s, but rarely beyond the fifth decade of life [5,11-13]. In our study, the rate of coexistence of PRAF and LDH was slightly higher than in previous reports by various other authors. PRAF with LDH is probably more common in adults than is generally recognized. It is difficult to capture bone fragments with X-ray and MRI. Therefore, CT is essential for imaging of PRAF [3,14]. In the present case series, one patient was diagnosed with PRAF based on postoperative CT despite the fact that preoperative X-ray and MRI showed no obvious bone fragments, and the surgeon had confirmed intraoperatively that no free hernia was present on the cephalad and medial sides (Figure 1).

Because of the rarity of PRAF with LDH, a consensus on the treatment strategy has not been reached. Surgery is the treatment of choice for lumbar buttock pain and leg pain refractory to conservative treatment. There is no consensus on whether decompression requires herniorrhaphy alone, simultaneous fragment removal, or extensive laminectomy with fusion. However, the treatments available for PRAF with LDH have not yet been fully investigated [5,15-20]. Shirado et al. [17] reported that good results can be obtained with herniotomy alone and that bone fragment resection is not mandatory. By contrast, Epstein and Epstein [2] and Epstein [4] reported that movable bone fragments are involved in neurological symptoms and that bone fragment resection is necessary because nerve root impingement is not adequately resolved. Additionally, Savini et al. [12] stated that treatment should be combined with fusion for iatrogenic instability due to extensive laminectomy and facetectomy for wide-based fragments.

A CT scan is desirable to improve the diagnosis rate, surgical planning, and intraoperative decision-making. Our current strategy is to first perform preoperative CT measurements to determine the position of the bone fragments and discs and measure the width of the lamina window, then confirm intraoperatively nerve decompression and bone fragment mobility, and finally determine the need for bony fragment removal based on the mobility of the nerve root after hernia removal and the instability of the bony fragment. Several options are available if bone resection is required. Bone fragment removal has been attempted under microscopy, the microendoscopic discectomy (MED) system, and, more recently, fully endoscopic spine surgery (FESS). Because the goal of surgery is to obtain adequate nerve decompression, it is essential to plan for bony resection sufficient to achieve that goal. If removal of the bony component is necessary, it is important to ensure an adequate visual field and working space. Some authors have suggested that bilateral or total discectomy is necessary for central lesions with large, wide basal bone fragments. We believe that in some cases, endoscopy can be utilized to remove the dissected bone fragments beyond the midline with unilateral entry. Matsumoto et al. [21] reported that MED is more feasible for patients with a lateral type of fragment. They treated PRAF with MED and reported good results, achieving a cure in 18 patients with PRAF. Patients with unilateral and central-type bone fragments were also compared in their study. The results indicated that the mean recovery rate of the JOA score was significantly higher in patients with unilateral than central fragments, with a mean follow-up of 21.1 months. The authors argued that the MED technique, which facilitates the removal of lateral bone fragments and nerve root retraction, is more effective in patients with lateral fragments. The MED technique is minimally invasive but requires appropriate patient selection and a skilled surgical technique [21]. Okada et al. [22] reported that it is possible to resect bone fragments using a unilateral or bilateral approach, even those with a wide base, using a curved Kerrison rongeur or chisel. FESS can be approached close to the midline of the symptomatic side, and simultaneous contralateral decompression is possible from one side. Therefore, it is a useful option for the removal of bone fragments. Few publications have focused on the application of FESS. Zuo et al. [23] reported good results in 9 of 57 cases of disc herniation associated with angle dissection that had ossified fragments, and they performed total removal of the fragments using FESS. We performed FESS and intraoperatively found strong tension after hernia removal in some cases, which allowed for good bone fragment processing.

Limitations

This study had several limitations. First, the surgical technique and treatment of bone lesions were chosen at the discretion of the attending physician. In reality, because PRAF is not common, what treatment modality is best remains controversial. The different physiological characteristics of younger and older patients may have influenced the surgical technique. Second, preoperative CT scans were not performed in all cases. The use of preoperative CT has recently decreased. The reasons for this trend include the decreased need for CT myelography because of improved MRI resolution and the risks of contrast agents and CT radiation exposure. Although younger patients are more conscious of avoiding CT exposure, it is desirable to perform CT at the level of the lower lumbar spine (the site of predilection) by narrowing the CT imaging range. CT-like MRI, which has been the focus of much attention in recent years, may help to solve this problem. Third, this was a single-hospital retrospective observational study, and the small number of patients may have influenced the clinical outcomes. Prospective studies involving multiple centers and large numbers of patients are desirable.

## Conclusions

We evaluated the coexistence rate of PRAF with LDH and the surgical outcomes at our hospital. The coexistence rate was 10.5%; thus, it is not rare. The outcomes were generally good. Although there is still no consensus on the treatment strategy for PRAF, we believe that some patients require bone fragment treatment. When bone resection is necessary, it is preferable to have a bone fragment treatment option. A CT scan is recommended to improve the diagnostic rate of PRAF and to assist in surgical planning and intraoperative decision-making.
